# Objective sleep characteristics of elite academy soccer players: a seven-day actigraphy analysis of a U15 national league squad

**DOI:** 10.5114/biolsport.2026.158301

**Published:** 2026-03-09

**Authors:** Lorcán Mason, James Connolly, Lydia E. Devenney, Karl Lacey, Maria Faulkner, Rónán Doherty

**Affiliations:** 1 Department of Sport Science and Performance, Atlantic Technological University Donegal, Port Road, Letterkenny, Donegal, Ireland, F92 FC93; 2 School of Computing, Engineering and Intelligent Systems, Ulster University, Northland Rd., Londonderry/Derry, UK, BT48 7JL; 3 School of Psychology and Counselling, The Open University, Walton Hall, Milton Keynes, UK, MK7 6AA

**Keywords:** Sleep, Athletes, Actigraphy, Adolescents, Youth

## Abstract

This study quantified objective sleep characteristics in elite academy soccer players using actigraphy and determined how day type (training days, non-training days, and competition days) influences sleep parameters. Objective sleep characteristics of elite academy soccer players (n = 13) were analysed across training days (TD), non-training days (NTD), and competition days (CD) using 7-day actigraphy monitoring. Significant differences were found between day types for total sleep time (TST) (χ^2^(2) = 8.59, p = 0.01, η^2^ = 0.09) and time in bed (TIB) (χ^2^(2) = 9.27, p = 0.01, η^2^ = 0.10). Training days yielded substantially lower values (TST: 431 ± 95.2 min; TIB: 478 ± 100 min) compared to non-training days (TST: 541 ± 231 min; TIB: 596 ± 240 min), representing mean differences of 110 minutes (95% CI: 39–181 min) for TST and 118 minutes (95% CI: 44–192 min) for TIB. No significant differences were found for sleep onset latency (SOL: 19.3 ± 19.6 min), number of awakenings (NoA: 39.8 ± 14.5), wake after sleep onset (WASO: 53.3 ± 25.8 min), or sleep efficiency (%SE: 84.4 ± 5.97%) (all p > 0.05). Critically, while TST and %SE met age-specific recommendations, NoA and WASO substantially exceeded thresholds for good sleep quality. Despite adequate sleep duration, elevated WASO and NoA indicate compromised sleep quality through fragmentation. These findings highlight the necessity for comprehensive sleep monitoring in elite adolescent athletes extending beyond duration alone. Future research should investigate targeted interventions addressing sleep fragmentation within the constraints of training, competition, and academic schedules.

## INTRODUCTION

Sleep is viewed as essential to the recuperation process for athletes [1, 2] and is a vital component in recovery through its involvement in growth, repair, regeneration, and immunity [1, 2]. Furthermore, the literature has shown that the sleep of athletes impacts elements of athletic performance including both physical and cognitive performance, recovery, injury risk, and mental well-being [1, 3]. The relationship between sleep and recovery in athletes can be viewed in terms of three key factors that affect restoration processes: 1. sleep duration (total sleep requirements including napping); 2. sleep quality (total sleep absent of sleep disorders, environmental disturbances, or sleep fragmentation); 3. sleep phase (circadian timing of sleep during the light-dark cycle) [4]. During adolescence, the psychosocial and societal pressures experienced such as academic and sporting pressures or their social environment, may result in adverse sleep health which negatively impacts the recuperation process [5, 6]. Sleep health is characterised by its individualised and multifaceted view of sleep-wake cycles, which considers an individual’s sleep duration and quality within an environmental and social context to promote wellbeing [7]. As such, sleep deprivation (an insufficient sleep duration compared to the basal level) and disturbances (the inability to initiate and/or maintain the sleep-cycle) are considered risk factors for adverse health, recovery, and injury risk in athletic populations [1, 4, 8–10].

**FIG. 1:**
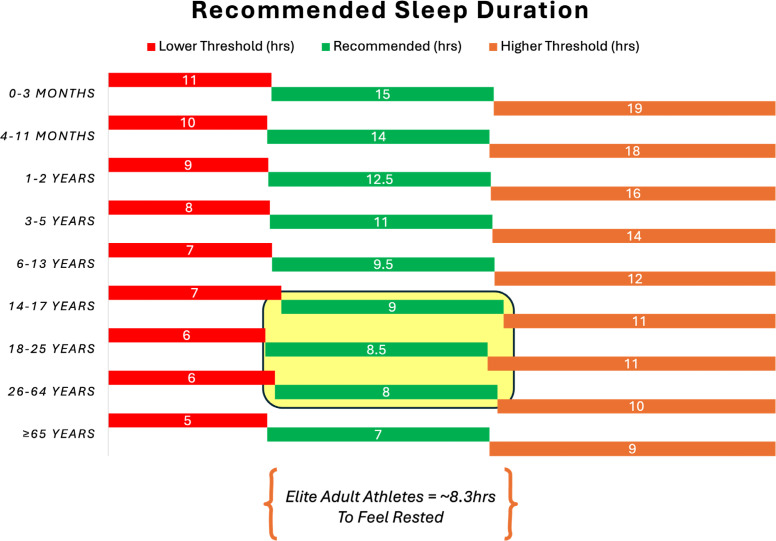
Recommended sleep durations for different age groups. Data compiled and adapted from [6, 11, 22]. Figure created by authors for this manuscript.

Sleep must align with an individual’s sleep need, age-dependent sleep duration and be of sufficient quality, to elicit sleeps restorative effects on the body [11]. Sleep need refers to the optimal amount of sleep necessary to sustain alertness and cognitive functioning during daily activities and athletic performance [1, 12]. Sleep duration can be affected by both external and internal environmental factors [1, 13, 14]. Substantial inter-individual variations exist, creating challenges in defining an ‘ideal’ sleep schedule [13–15]. These variations emphasize the multifaceted nature of sleep regulation and its impact on overall well-being [1, 16]. Research indicates that athletes may have higher sleep requirements than the general population [1, 12, 17]. Hence, the recommended guidelines (figure 1) may not suffice for athletes or adolescents, thus, a more personalized strategy considering overall sleep quality could be more suitable for addressing an individual athlete’s sleep need [1, 17]. Elite athletes typically require approximately 8.3 ± 0.9 hours of sleep to feel adequately rested [12, 18], while 8–11 h are recommended for adolescents [11]. However, research has demonstrated that adolescent athletes achieve 6–7 hours of sleep on average [19–21], highlighting a disparity between current guidelines and actual sleep durations [6, 11, 22]. During adolescence, there are changes in sleep-wake patterns as part of the developmental process, leading to adjustments in how sleep is regulated during this period [22]. These modifications in sleep regulation enhance the ability to tolerate sleep pressure by reducing adenosine accumulation [5, 6, 23] and are influenced by the maturation of physiological, psychological, and cognitive functions, and a delayed circadian rhythm [24], thus creating conditions for insufficient sleep due to external factors related to adolescence [5, 6, 24]. Consequently, the amount of time spent asleep decreases steadily between the ages of 15 and 18, with studies showing a reduction of approximately 1.5 to 3 hours during adolescence [20, 23]. Despite this trend, the sleep requirements of adolescents under normal living conditions appear to meet current recommendations ~9.25 h [6], notwithstanding of their maturation status [25], suggesting that the decline in sleep duration is more influenced by environmental factors rather than biological factors [23, 24].

Furthermore, elite youth sports present an additional challenge for adolescents as a result of the high volume of training, heightened intensity of training, and rigorous competition schedules [10, 20, 26]. Consequently, increased involvement in elite sports may heighten the risk of musculoskeletal injuries [27–29]. As such, the prevalence of injuries among adolescent athletes has risen in recent times [30], with incidence rates ranging from approximately 1.4 to 6.4 per 1000 hours and 22.4 per 1000 hours during training and competition respectively [29, 31]. Therefore, the identification of modifiable risk factors which aid recovery and reduce the risk of injuries is crucial [2, 8, 32–34].

Both sleep and nutrition are viewed as modifiable facilitators for recovery in athletes [1, 2, 35], thus emphasising their relevance to injury mitigation interventions. Research indicates that adolescent athletes who achieve < 8 h of sleep per night are 1.7 times (95% CI; 1.0–3.0; p = 0.04) more likely to sustain an injury [8]. Furthermore, during periods of intense training, decreasing sleep durations results in a 2.25-times increase (95% CI; 1.46–3.45; p < 0.001) in injury risk [34]. Moreover, a reduction in sleep duration alone was associated with a 1.46-fold higher risk of injury (95% CI; 1.10–1.94; p < 0.01) increased risk, while adolescent athletes who specifically reported sleeping less than 8 hours had a 1.31-fold (95% CI; 0.97–1.78; p = 0.080) increased injury risk [34]. Furthermore, actigraphy research has indicated that Wake after sleep onset (WASO), which represents sleep disruption, as a predictor of previous injury (OR = 1.144), whilst time spent awake (TA) was found to predict injury occurrence (OR = 0.974) [19]. Moreover, adolescent athletes who decreased their TA by at least 1 min reduced their risk of future injury ([F_(2.36)_ = 6.512; p = 0.004]) [19].

Actigraphy has emerged as a practical validated tool for objectively assessing sleep-wake patterns in athletic populations, offering continuous monitoring capability that overcomes the limitations of subjective self-reporting measures [17, 36–39]. Current actigraphy research in adolescent athletes has demonstrated variable sleep patterns, with research reporting average sleep durations of ~6.3 hours in youth athletes [40], substantially lower than the recommended guidelines. Furthermore research has identified specific sleep parameters, WASO (OR = 1.144) and time awake (OR = 0.974) as predictors of musculoskeletal injury in adolescent athletes [19], establishing the clinical relevance of objective sleep monitoring in adolescent athletes. However, limited research has systematically examined how sleep parameters measured via actigraphy vary across different training and competition contexts within elite adolescent soccer athletes, creating a gap in the understanding of the temporal characteristics in relation to athletic demands.

Despite the established associations between sleep, athletic performance, and injury risk, limited research has employed objective measurement techniques to characterise the sleep patterns of elite adolescent soccer players across different training and competition contexts. Understanding how sleep parameters vary between training days, non-training days, and competition days is critical for identifying periods of compromised recovery and informing targeted intervention strategies. Therefore, this study aimed to: (1) quantify the objective sleep characteristics of elite academy soccer players using actigraphy over a 7-day monitoring period; and (2) determine whether sleep duration and quality parameters differ significantly between training days, non-training days, and competition days. Such knowledge may enable practitioners to implement evidencebased sleep hygiene strategies tailored to the specific demands of adolescent elite soccer training and competition schedules.

## MATERIALS AND METHODS

### Participants

Convenience sampling techniques were utilised during this study, as the population of interest matched a specified criteria which may not be met in the general population [41]. Participants (n = 13) were recruited from an elite soccer academy of a League of Ireland club. The participants characteristics are presented in (Table 1) and were defined as elite by the following: (a.) currently receiving support/funding through the international carding scheme and/or (b.) members of a national/professional team or a recruitment/academy squad and/or (c.) nationally ranked in their sport [42]. Participants were excluded if they were (a.) aged > 18 years (b.) were classified < Tier 3 on the participant classification framework [42] or (c.) had a diagnosed sleep disorder. Three participants were excluded from analysis due to noncompliance with the instructed protocols.

**TABLE 1:** Participant characteristics n = 13 (means ± standard deviations (SD)).

	Mean ± SD
Age (years)	14.4 ± 0.51
Height (cm)	173.1 ± 9.5
Body mass (kg)	62.8 ± 6.05
Hours spent in training and or competition per week (mins)	472.3 ± 155.79
TST (min)	505 ± 192
TIB (min)	558 ± 203
SOL (min)	19.3 ± 19.6
WASO (min)	53.3 ± 25.8
%SE	84.4 ± 5.97
NoA	39.8 ± 14.5
WBD (min)	1.38 ± 0.59

TST (min) = Total Sleep Time; TIB (min) = Time in Bed; SOL (min) = Sleep Onset Latency; WASO (min) = Wake After Sleep Onset; %SE = % Sleep Efficiency; NoA = Number of Awakenings; WBD (min) = Average Wake Bout Duration; TD = Training Day; NTD = Non-Training Day; CD = Competition Day.

### Procedure

**FIG. 2:**
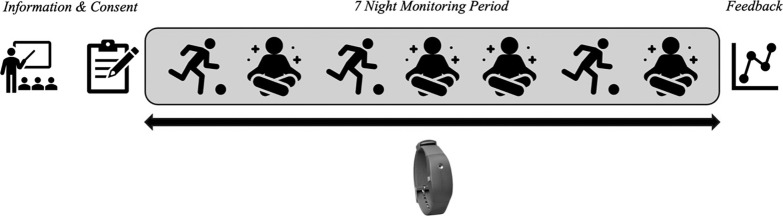
Schematic of actigraphy monitoring period.

All procedures were approved by the research ethics committee of the Faculty of Business of the University and Garda Vetting was acquired by the principal investigator prior to data collection. After reading the participant information sheet, the athletes’ parent/ guardian(s) were invited to provide informed consent via Qualtrics^xm^. Participants were then required to provide consent and were given information on the use of actigraphy and were instructed on how to wear and care for the actigraphy device. Each participant was given an ActiWatch 2 (Philips Respironics ActiWatch 2, Phillips, Amsterdam, Netherlands), and instructions were provided. Participants were instructed to wear the ActiWatch 2, with the exception of bathing, for a period of 7-days during the mid-season competitive period. This phase of the season represents a typical; in-season microcycle characterised by habitual training frequency (2–3 training sessions), one scheduled match day, and at least one non-training recovery day. This selection criterion ensured that data collection reflected normative training and competition loads rather than atypical periods such as pre-season intensification, fixture congestion, or competition-free phases [1, 13, 43, 44]. For analytical purposes, each sleep episode was classified according to the preceding day’s activity type: training days (TD; sleep following days involving scheduled team training sessions), non-training days (NTD; sleep following complete rest or recovery days without organised training), and competition days (CD; sleep following days on which official competitive matches occurred). This classification approach, categorising sleep episodes by the preceding day type, enables examination of how different training and competition demands influence subsequent sleep/wake patterns. Following completion of the study all participants received feedback and a summary of their data (Figure 2).

#### Actigraphy

Actigraphy measurement were also utilised during this research which involved a small wrist-worn device (Actiwatch 2; Philips Respironics, Eindhoven, Netherlands) which monitors time weighted whole-body motion [17]. Actigraphy is a reliable sleep measurement tool validated against PSG [36] for the purpose of measuring sleep with a 93% sensitivity and an 87% accuracy in sleep-wake cycle assessment [37–39]. Average objective sleep characteristics were calculated for any obtained major sleep episode [45], defined as the primary nocturnal sleep period. Whilst actigraphy devices continuously monitor activity, the present analysis focused exclusively on nocturnal sleep parameters; daytime napping episodes were not systematically identified or quantified. Limitations exist with actigraphy for sleep assessment as all activity is classified as wakefulness unless activity measures reach a supressed threshold inferring a prone position [46].

The data collected was then used to calculate indicators of sleep continuity including total time in bed (TIB), total sleep time (TST), sleep onset latency (SOL; time from light out to N1), wakefulness after sleep onset (WASO; amount of time awake after sleep onset), number of awakenings (NoA) and sleep efficiency (%SE; ratio of TST:TIB) [47]. For sleep to be regarded as good quality, the current recommendations suggest values of ≤ 15 minutes for SOL, ≥ 85% for SE and ≤ 20 minutes WASO [19, 48].

### Data Analysis

Descriptive and inferential statistics were utilised for analysis [41], using excel for data cleaning and Jamovi version 2.3 (The Jamovi Project, Sydney, Australia) for analysis. Descriptive statistics and frequency distribution were used to present findings [41]. Data normality and distribution were assessed using histograms, Q-Q plots and the Shapiro-Wilks test [41]. If the assumptions of normality were met, differences between the TD, NTD and CD were explored by performing a one-way ANOVA [41]. The differences between nonnormal data were assessed by Kruskal-Wallis one-way ANOVA [41]. Descriptive analysis facilitated the observation of the general characteristics of the sample in the form of central tendencies and variability [41, 49]. Inferential analysis facilitated the understanding of relationships, differences and cause and effect between variables collected [41, 49]. Data are reported as mean ± standard deviation (SD), unless otherwise stated. The alpha level was set to P ≤ 0.05 to determine significance unless otherwise stated. Effect size (ES) for pairwise comparison were calculated using eta squared (η^2^) and were classified as follows: trivial (ES: < 0.20), small (0.21–0.60), moderate (0.61–1.20), large (1.21–2.00), or very large (> 2.00) [50].

## RESULTS

### Objective Sleep Characteristics

**FIG. 3:**
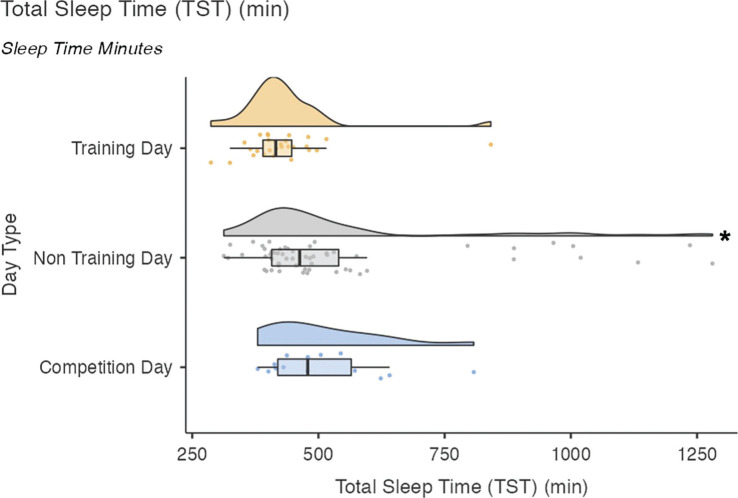
Total Sleep Time (TST) minutes by day type (*p = 0.022).

**FIG. 4:**
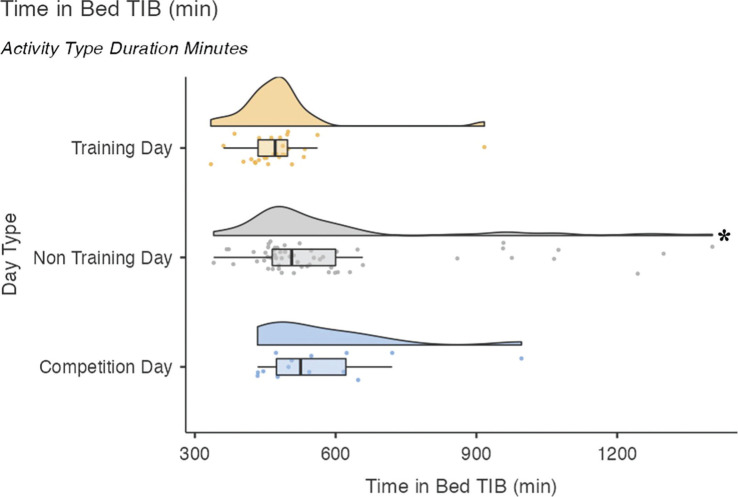
Time in Bed (TIB) minutes by day type (*p = 0.014).

The objective characteristics for TD, NTD, and CD are presented in Table 2. Significant differences were determined between days for measures of Total Sleep Time (TST) (χ^2^(2) = 8.59, p = 0.01, η^2^ = 0.09, small effect) with mean differences of 110 minutes between TD and NTD (95% CI: 39–181 min), and for Time in Bed (TIB) (χ^2^(2) = 9.27, p = 0.01, η^2^ = 0.10, small effect) with mean differences of 118 minutes between TD and NTD (95% CI: 44–192 min). Mean TST was 431±9 5. 2 min for TD, 541±231 min for NTD, and 509±119 min for CD (Figure 3). Mean TIB was 478 ± 100 min for TD, 596 ± 240 min for NTD, and 569 ± 151 min for CD (Figure 4). For measures of Sleep Efficiency (%SE), no significant differences were found between days as determined by one-way ANOVA (F_(2,34.4)_ = 0.99, p = 0.38, η^2^ = 0.05, small effect), with mean %SE of 83.2 ± 5.78 for TD, 85.1 ± 6.08 for NTD, and 84 ± 5.85 for CD.

**TABLE 2:** Objective Sleep Characteristics (n = 13) (means ± standard deviations (SD)).

	TD	NTD	CD
TST (min)	431 ± 95.2 [95% CI: 393.9–467.7]	541 ± 231 [95% CI: 479.0–602.5]	509 ± 119 [95% CI: 440.4–577.9]

TIB (min)	478 ± 100 [95% CI: 438.7–516.4]	596 ± 240 [95% CI: 531.4–659.8]	569 ± 151 [95% CI: 481.9–656.2]

SOL (min)	22.2 ± 14.6 [95% CI: 16.6–27.9]	18.4 ± 22.5 [95% CI: 12.4–24.5]	17.0 ± 15.8 [95% CI: 7.8–26.1]

WASO (min)	46.7 ± 19.7 [95% CI: 39.1–54.3]	54.9 ± 22.1 [95% CI: 49.0–60.8]	59.9 ± 44.0 [95% CI: 34.5–85.3]

%SE	83.2 ± 5.78 [95% CI: 81.0–85.5]	85.1 ± 6.08 [95% CI: 83.5–86.8]	84.0 ± 5.85 [95% CI: 80.6–87.4]

NoA	36.4 ± 11.1 [95% CI: 32.1–40.7]	40.1 ± 12.6 [95% CI: 36.7–43.5]	45.9 ± 23.9 [95% CI: 32.0–59.7]

WBD (min)	1.24 ± 0.45 [95% CI: 1.07–1.42]	1.44 ± 0.62 [95% CI: 1.27–1.60]	1.41 ± 0.69 [95% CI: 1.01–1.80]

TST (min) = Total Sleep Time; TIB (min) = Time in Bed; SOL (min) = Sleep Onset Latency; WASO (min) = Wake After Sleep Onset; %SE = % Sleep Efficiency; NoA = Number of Awakenings; WBD (min) = Average Wake Bout Duration; TD = Training Day; NTD = Non-Training Day; CD = Competition Day.

No significant differences were observed for Sleep Onset Latency (SOL) as determined by Kruskal–Wallis one-way ANOVA (χ^2^(2) = 3.88, p = 0.14, η^2^ = 0.04, trivial effect), with mean mean SOL of 22.2 ± 14.6 min for TD, 18.4 ± 22.5 min for NTD, and 17.0 ± 15.8 min for CD. Similarly, no significant differences were found for Number of Awakenings (NoA) (χ^2^(2) = 2.96, p = 0.23, η^2^ = 0.03, trivial effect), with means of 36.4 ± 11.1, 40.1 ± 12.6, and 45.9 ± 23.9 for TD, NTD, and CD respectively. Moreover, for Wake After Sleep Onset (WASO), no significant differences were observed between days (χ^2^(2) = 3.79, p = 0.15, η^2^ = 0.04, trivial effect), means of 46.7 ±19.7 min for TD, 54.9 ± 22.1 min for NTD, and 59.9 ± 44.0 min for CD.

## DISCUSSION

The aims of this research were to examine the objective sleep characteristics of a cohort of elite academy soccer players using actigraphy measures. Furthermore, this research aimed to examine the differences between sleep characteristics on both training and nontraining days and days of competition. The key results were that significant differences were found between day type (TD, NTD and CD) for TST and TIB. Furthermore, no significant differences were found between day type (TD, NTD and CD) for measures SOL, NoA, WASO, and %SE. Moreover, SOL, TST, and %SE met the age-specific recommendations, however, NoA, and WASO were outside the recommended values for appropriate sleep duration and quality [11, 48]. Although players achieved the recommendations for sleep duration, their quality of sleep was unsatisfactory, thus, strategies to improve sleep quality measures in this cohort are recommended.

The finding of this research suggest that this cohort of elite academy soccer players achieved the National Sleep Foundations age-specific recommendations for TST (Figure 3) (505 ± 192 min) [11]. TST exceeded previous findings which suggested that elite athletes need ~498 min of sleep to feel rested [12, 18] and also exceeded previous findings, which stated an average TST of ~378 min in adolescent athletes [6, 22]. These findings are in contrast to the general consensus which suggests that adolescents may present with insufficient TST below recommendations (90–180 min) [19–21]. Furthermore, as research has suggested that adolescent athletes who achieve ≤ 480 min of TST per night are 1.7 times (95% CI; 1.0–3.0; p = 0.04) more likely to sustain an injury [8], the achieved TST may act as a positive modifiable factor against injury in this cohort. This is further evidenced from research which suggested that adolescent athletes who slept for more than 480 min per weeknight decreased their injury risk by 61% (OR, 0.39; 95% CI, 0.16–0.99) [51]. To note, this study categorised all competition-associated sleep episodes collectively as ‘competition days’ (CD), without differentiating between pre-match (MD-1) and post-match (MD+1) sleep. Prior research has demonstrated that pre-competition sleep may be particularly disrupted due to performance anxiety and psychological arousal, whilst postcompetition sleep may be affected by physical exertion, delayed bedtimes, and scheduling demands [1, 16]. The absence of time-bound variation in our analysis may have masked distinct sleep patterns; future research with larger sample sizes should examine MD-1 and MD+1 sleep separately to explain how anticipatory and recovery processes distinctly influence sleep architecture. However, the observed variations in TST and TIB across different day types align with emerging evidence regarding the influence of training and competition demands on sleep patterns in soccer players [1, 13, 43, 44]. Recent research has examined how different training session characteristics affect professional soccer players’ sleep, demonstrating that both training intensity and training type significantly impact nocturnal sleep duration and architecture [44]. These findings support our observation that TDs are associated with reduced sleep opportunity (TIB) compared to NTDs, likely reflecting the competing demands on adolescent athletes’ time, including academic commitments, social activities, and structured training schedules [1, 6, 13, 43, 44]. Furthermore, the elevated TST and TIB on NTDs observed in our cohort may represent a compensatory ‘sleep extension’ response, whereby athletes naturally increase sleep duration when training demands are reduced [1, 6, 13, 18, 44]. This pattern, whilst potentially beneficial for addressing accumulated sleep debt, underscores the challenge of maintaining consistent sleep-wake schedules across different day types, a key component of optimal sleep hygiene [1, 52].

In contrast to TST findings, NoA, and WASO failed to meet the recommendations advised for this cohort [11, 48]. Previous research has stated that NoA ≥ 3 does not indicate good sleep quality in adolescents [48] which contrasts with the 39.8 ± 14.5 found in this cohort of athletes. Furthermore, WASO (53.3 ± 25.8) was found to greatly exceed the recommended ≤ 20 minutes [48], which coupled with the excessive NoA may indicate poor sleep quality in this cohort of athletes, thus, potentially impeding improvements in athletic performance [3] and an increased injury risk [8]. Beyond environmental and societal factors, behavioural and psychological stressors associated with elite adolescent athletic participation may contribute substantially to sleep fragmentation [10, 13, 20, 37, 53]. Adolescent athletes navigate multiple concurrent demands including academic performance expectations, athletic development pressures, social relationship maintenance, and maturational changes, creating a psychosocial context conducive to cognitive arousal and worry [5, 6, 20]. Pre-sleep cognitive activity, particularly worry about upcoming competitions or rumination regarding performance, can prolong sleep onset latency and increase nocturnal awakenings [1, 54, 55]. Sleep optimisation strategies should therefore extend beyond purely behavioural sleep hygiene recommendations to encompass psychological skills training [1, 17, 18, 52]. Evidence-based approaches may include: (1) establishing consistent pre-sleep routines incorporating relaxation techniques (e.g., progressive muscle relaxation, controlled breathing); (2) cognitive-behavioural strategies for managing performance-related anxiety; (3) sleep environment optimisation (cool temperature, minimal light and noise); (4) strategic scheduling of academic and training demands to reduce evening cognitive load; and (5) education regarding appropriate timing of stimulant consumption (caffeine restriction 8–9 hours pre-bedtime) [1, 17, 18, 52].

Therefore, strategies to improve sleep quality including sleep hygiene and education interventions [18, 52] may be justified in this cohort. It must be noted, the present study did not systematically control for behavioural and environmental factors known to influence sleep quality, including pre-sleep screen exposure (blue light), caffeine consumption timing, or bedroom environmental characteristics (temperature, light, noise) [1, 35, 56]. Emerging evidence suggests that evening smartphone use significantly impairs sleep quality and next-day performance in elite soccer players [54, 55], whilst caffeine consumption in the hours preceding sleep can substantially increase sleep onset latency and reduce sleep efficiency [57]. However, recent actigraphy research conducted in adolescent athletes found that increased WASO is predictor of previous injury (OR = 1.144), while an increased time spent awake was found to predict injury occurrence (OR = 0.974) [19]. Furthermore, researchers found that adolescent athletes who decreased their time spent awake per night by ≥ 1 min, reduced their likelihood of sustaining future injury ([F_(2.36)_ = 6.512; p = 0.004]) [19].

### Limitations

A major strength of this case study was the use of objective measures in the form of actigraphy, for the assessment of sleep over a 7-day period, however, this case study is not without limitations. Firstly, the sample size used for this investigation was limited due to access to participants and compliance. As such, the generalisability of these findings warrants consideration. Participants were recruited from a single elite League of Ireland academy using convenience sampling, representing a specific geographical, cultural, and competitive context. Factors such as coaching philosophies, training scheduling, and support infrastructures (e.g., access to sports science, nutrition, and sleep education resources) may differ substantially across academies, leagues, and nations [10, 58–60]. Whilst the participant classification framework employed [42] ensures that athletes meet objective elite status criteria, caution should be exercised when generalising findings to elite youth soccer populations operating under different organisational or cultural contexts [10, 60, 61]. Nevertheless, the observed results, adequate sleep duration but compromised sleep quality, may represent a generalisable phenomenon in adolescent elite athletes, given the universal biological changes in sleep regulation during adolescence and the common demands of balancing elite sport with academic and social development [5, 6, 20]. Additionally, as this was a practical investigation, actigraphy rather than the gold-standard measurement of sleep, Polysomnography (PSG), was used due to the access and availability of equipment. Moreover, the lack of subjective sleep measurement tools is also a limitation. Subjective measures of sleep can be an efficient and effective means of collecting sleep measures [1, 17, 62], and can aid in the validation of the objective data collected by offering a greater global assessment of sleep [1, 17]. Additionally, the present study focused exclusively on nocturnal sleep parameters and did not systematically quantify daytime napping behaviour. Adolescent athletes may utilise strategic napping to supplement nocturnal sleep, particularly following intensive training or competition [1, 63]. Future research should employ comprehensive 24-hour sleep assessment protocols that capture both nocturnal sleep and daytime napping to provide a complete picture of sleep behaviour in this population. A further limitation concerns the potential influence of match location on sleep parameters. The present study did not differentiate between home and away competition days; travel associated with away fixtures may introduce additional disruption to sleep timing and quality through factors such as altered sleep environment, delayed return home times, and travel-related fatigue [1, 10, 13, 64, 65]. Future research should systematically examine whether match location moderates the relationship between competition and subsequent sleep characteristics.

## CONCLUSIONS

This investigation provides novel, objective characterisation of sleep patterns in elite academy soccer players across training, non-training, and competition contexts. The primary finding was that total sleep time (TST) and time in bed (TIB) varied significantly according to day type, with TDs associated with substantially reduced sleep opportunity, approximately 110 minutes less TST, compared to NTDs. This pattern indicates that the structured demands of training, combined with academic and social commitments, markedly constrain adolescent athletes’ capacity for adequate sleep during competitive training periods, potentially necessitating compensatory sleep extension on NTDs.

In contrast, sleep quality measures, including sleep onset latency (SOL), wake after sleep onset (WASO), number of awakenings (NoA), and sleep efficiency (%SE), did not differ significantly across day types, suggesting that while sleep opportunity fluctuates with training demands, sleep quality remains consistently below recommendations [1, 10, 11, 48]. Although SOL, TST, and %SE met agespecific recommendations, elevated NoA and WASO values exceeded thresholds indicative of good sleep quality [16, 48, 53]. This dissociation of adequate sleep duration but compromised quality, represents a clinically meaningful finding with important implications for athlete recovery, performance, and injury risk.

From a practical standpoint, these findings highlight the necessity for comprehensive sleep monitoring frameworks in elite adolescent athletes that extend beyond the assessment of sleep duration alone [17, 37, 66]. Routine inclusion of objective and subjective sleep quality metrics, particularly indicators of sleep fragmentation such as NoA and WASO, should be prioritised to identify athletes at heightened risk of impaired recovery and potential injury [1, 17, 37, 66]. In addition, multi-component, evidence-based sleep hygiene interventions tailored to the developmental and competitive demands of this population are warranted. Effective strategies may include maintaining consistent sleep-wake schedules to support circadian stability, enforcing screen curfews of ≥ 2 hours before bedtime to limit blue light exposure and cognitive stimulation, restricting caffeine intake to ~8–9 hours before sleep, and optimising environmental factors such as temperature (cool), light (darkened), and noise control [1, 18, 52]. Furthermore, organisational consideration of training, academic, and competition schedules is essential to preserve adequate sleep opportunity throughout the season.

From a research perspective, further investigation is warranted to outline the complex interaction between sleep opportunity, sleep quality, and adolescent development. Future studies should employ longitudinal, multi-centre designs with larger sample sizes to enhance generalisability, and integrate both objective (actigraphy, polysomnography) and subjective sleep assessments to provide a comprehensive characterisation of sleep. Additionally, studies should differentiate between pre-competition and post-competition sleep, systematically monitor behavioural sleep hygiene practices and psychosocial stressors, and explore dose-response relationships between sleep interventions and key athletic outcomes, including performance, recovery, well-being, and injury incidence.

In conclusion, while these elite adolescent soccer players achieved recommended sleep durations, their consistently poor sleep quality underscores the need for systematic monitoring, education, and intervention. Multi-faceted approaches that address behavioural, environmental, and organisational factors are essential to optimise sleep, enhance recovery, and support the holistic development and performance of young athletes.
